# Endometrial Stromal Sarcomas: A Revision of Their Potential as Targets for Immunotherapy

**DOI:** 10.3390/vaccines6030056

**Published:** 2018-08-25

**Authors:** Sandra Tuyaerts, Frédéric Amant

**Affiliations:** 1 Division of Gynecologic Oncology, Department of Oncology, KU Leuven, 3000 Leuven, Belgium; frederic.amant@uzleuven.be; 2 Leuven Cancer Institute (LKI), 3000 Leuven, Belgium; 3 Division of Gynecology & Obstetrics, UZ Leuven, 3000 Leuven, Belgium; 4 Center for Gynecologic Oncology Amsterdam (CGOA), Antoni Van Leeuwenhoek-Netherlands Cancer Institute (Avl-NKI) and University Medical Centra (UMC), 1066 CX Amsterdam, The Netherlands

**Keywords:** endometrial stromal sarcoma, recurrent chromosomal translocations, neoantigen, immunotherapy

## Abstract

Endometrial stromal sarcomas are a subtype of uterine sarcomas that are characterized by recurrent chromosomal translocations, resulting in the expression of tumor-specific fusion proteins that contribute to their tumorigenicity. These characteristics make the translocation breakpoints promising targets for immunotherapeutic approaches. In this review, we first describe the current knowledge about the classification of endometrial stromal sarcomas, and their molecular and genetic characteristics. Next, we summarize the available data on the use of translocation breakpoints as immunotherapeutic targets. Finally, we propose a roadmap to evaluate the feasibility of immunologic targeting of the endometrial stromal sarcoma-specific translocations in patients with recurrent disease.

## 1. Introduction

Endometrial stromal sarcomas (ESS) are translocation-related sarcomas that are part of the uterine sarcoma family [1,2]. ESS are divided into two subtypes, based on their grade. Low-grade ESS (LG-ESS) follow an indolent growth pattern and respond quite well to hormonal therapy. However, late relapses are very frequent, emphasizing the need for therapeutic options to prevent relapse. Adjuvant immunotherapy using vaccines targeting the translocation breakpoint fusion protein could be useful to prevent relapse, because vaccination induces immunological memory [3,4]. When the tumor antigen (translocation breakpoint) becomes visible again to the immune system, the memory T cells will be present to attack the tumor cells. The late relapse phenomenon also urges for the identification of a good biomarker to detect disease relapse early, before it becomes radiographically measurable. The translocation breakpoint fusion protein could also play a role as a biomarker. Recently, novel genomic analysis techniques have been developed to identify chromosomal translocations. While these chromosomal translocations were originally identified by G-banding karyotyping and fluorescence in situ hybridization (FISH), newer methods are long-range multiplex (digital droplet) polymerase chain reaction (PCR) and next-generation sequencing (NGS). These novel techniques allow the detection of the tumor-specific breakpoint in cell-free tumor DNA (cf-DNA). Recent preliminary data indicate that these techniques can be developed as a personalized liquid biopsy for patient follow-up [5,6,7,8].

High-grade ESS (HG-ESS) on the other hand are generally detected in a more advanced stage, with a higher tumor burden. For these tumors, no adequate adjuvant therapy is available. Immunotherapy might also play a role here, using the transfer of adoptive T cells targeting the translocation breakpoint fusion protein. Adoptively transferred T cells have been shown in other tumor types to be capable of eradicating large tumor burdens, and to persist as long-lasting memory cells in the blood to tackle relapse [9].

Herein, we summarize the most recent data of the clinical characteristics of ESS, and we describe in detail the different chromosomal translocations that have been described. We focus on the immunogenicity of translocation breakpoint antigens, and immunotherapeutic studies targeting these breakpoints, in order to provide a roadmap for the evaluation of immunotherapy feasibility in ESS.

## 2. Clinicopathological Characteristics and Treatment of Endometrial Stromal Sarcoma

ESS are rare tumors, comprising ~20% of all uterine sarcomas and <1% of all primary uterine malignancies. Currently, the WHO recognizes two subtypes of ESS, LG-ESS and HG-ESS, based on differences in morphology, molecular genetics, immunohistochemistry, and clinical behavior [1,2].

LG-ESS are the most common type and are diagnosed at a mean age of 50 years, with half of the patients diagnosed in the premenopausal stage. Obesity, diabetes, younger age at menarche, and tamoxifen intake have been associated with an increased risk of developing LG-ESS, although the mechanisms need to be elucidated. The main symptoms of LG-ESS are abnormal uterine bleeding and pelvic or abdominal pain. These tumors involve the endometrium, and infiltrate into the myometrium as irregular tongue-like structures, and show lymphovascular space invasion. The tumor cells are small cells with round to oval nuclei and scant cytoplasm, and usually show a low mitotic activity (<5 mitotic figures per 10 high power fields (HPFs)). LG-ESS tumors follow an indolent disease course and most cases (60%) present with stage I disease. Tumor stage is the most important prognostic factor, with a 5-year OS rate of >90% for stage I patients, while advanced stage tumors have decreased survival rates of 40–50%. However, the disease is characterized by late recurrences, even in patients with stage I disease, that can occur 10–20 years after the initial diagnosis. Thus, long-term follow-up of these patients is warranted. Relapses can be local (pelvic/vaginal) or distant (abdomen, lungs). Lymph node involvement is seen in ~30% of patients. The initial treatment is hysterectomy and bilateral salpingo-oophorectomy, but in younger patients, fertility-sparing treatment can be considered. Chemotherapy and/or radiotherapy for advanced stage disease are usually ineffective. Because the vast majority of LG-ESS (up to 100% in some reports) show hormone receptor expression (Table 1), endocrine therapy is believed to be the most effective treatment for recurrent or metastatic disease [1,10,11,12,13]. 

HG-ESS is a distinct clinicopathologic entity that was reintroduced in the 2014 WHO classification [1,10,11,12], but continues to evolve based on novel molecular insights. Tumors are composed of atypical cells resembling endometrial stromal cells, but lacking the degree of polymorphism required for the diagnosis of undifferentiated uterine sarcoma. The round cells are typically larger than those of LG-ESS and tend to form tight nests. HG-ESS often exhibit tongue-like myometrial invasion, and more than half of the tumors show spindle cell areas, which are of low-grade. Their mitotic activity is consistently higher (>10 mitoses per 10 HPFs) compared to LG-ESS. Besides the above-mentioned differences, both tumor types appear to be histologically quite similar. Little is known about the natural course of HG-ESS, but these patients typically present with advanced stage disease (FIGO stage II-IV) and show frequent relapses within a few years after diagnosis. Median OS for these patients ranges from 11 to 23 months. As for LG-ESS, treatment consists of hysterectomy and bilateral salpingo-oophorectomy. Currently, there is no proof that adjuvant radio- and/or chemotherapy leads to a survival benefit. The 2014 WHO classification states that HG-ESS typically harbor the *YWHAE-NUTM2* gene fusion, but novel findings uncovered another subtype with the *ZC3H7B-BCOR* gene fusion that mimic myxoid leiomyosarcomas morphologically. The majority of these tumors also have mitotic indices of >10 mitoses per 10 HPFs, and consist of haphazard fascicles of spindle cells embedded in variable amounts of myxoid matrix. These patients present at higher stage and have a worse prognosis compared to LG-ESS [2,14]. 

## 3. Molecular and Genetic Characteristics 

### 3.1. Immunohistochemical Characteristics

The immunohistochemical profiles of the different types of ESS tumors is tabulated in Table 1. LG-ESS are typically positive for CD10, estrogen receptor (ER) and progesterone receptor (PR), while HG-ESS carrying the *YWHAE-NUTM2* fusion do not express these molecules. These tumors instead express high levels of cyclin-D1, cKIT, and BCOR. HG-ESS that harbor the *ZC3H7B-BCOR* fusion on the other hand are CD10-positive and show variable staining for ER and PR. BCOR and cyclin-D1 are expressed in the majority of these tumors. BCOR expression is a highly sensitive marker to identify HG-ESS with the *YWHAE-NUTM2* fusion and *BCOR* internal tandem duplication (ITD), as well as a subset of *BCOR*-rearranged HG-ESS [12,15].

### 3.2. Genetic Characteristics

ESS tumors are generally characterized by recurrent chromosomal translocations, both in LG-ESS and HG-ESS. These chromosomal translocations result in the fusion of genes involved in epigenetic regulation, either through polycomb-mediated gene silencing or post-translational covalent modification of histone proteins, thereby driving the neoplastic transformation process [12]. Table 2 shows a summary of the translocations described to date in ESS tumors.

The most common translocation in LG-ESS is t (7; 17) (p15; q21) resulting in the *JAZF1-SUZ12* gene fusion and it occurs in ~50% of patients [16,17,18]. Recently, it has been shown that the JAZF1-SUZ12 fusion protein destabilizes the polycomb repressive complex 2 (PRC2), resulting in decreased histone methyl transferase activity and subsequent activation of chromatin/genes normally repressed by PRC2 [27]. Less frequent fusion proteins due to chromosomal translocations in LG-ESS are JAZF1-PHF1, EPC1-PHF1, PHF1-MEAF6 [20], MBTD1-CXorf67 [21], and JAZF1-BCORL1 [22]. These chromosomal translocations are generally found to be mutually exclusive. Up to now, the exact mechanism underlying these translocations is not understood. Although very poorly described, it appears that the unifying mechanism of oncogenesis in LG-ESS is transcriptional dysregulation by altering chromatin remodeling [12].

In the 2014 WHO classification, HG-ESS have re-entered the classification based on novel molecular findings. These tumors harbor a unique *YWHAE-NUTM2* gene rearrangement, giving rise to a 14-3-3ε oncoprotein [12,23,24]. Inhibition of expression of the fusion protein results in reduced cell growth and migration. The YWHAE-NUTM2 fusion protein maintains an intact 14-3-3ε oncoprotein which is directed to the nucleus by a NUTM2 nuclear localization sequence [24,28]. A recent publication showed that these tumors are particularly responsive to anthracycline-based therapy, pointing out the relevance of molecular testing to reach an optimal treatment decision [2,29]. 

In addition to *YWHAE-NUTM2* rearranged tumors, tumors carrying the ZC3H7B-BCOR fusion protein have also been proposed as a HG-ESS subtype [12,14,25]. *ZC3H7B-BCOR*-rearranged ESS should be distinguished from other myxoid uterine mesenchymal tumors, given the implications for patient management [14]. 

Finally, ITD of *BCOR* have recently also been proposed to identify a subtype of HG-ESS. Ongoing clinicopathologic and immunohistochemical studies will clarify whether these neoplasms are best classified as HG-ESS, or whether they represent a distinct variant of truly undifferentiated (i.e., nonendometrial stromal) uterine sarcoma [2,15,26]. 

## 4. Immunogenicity of Translocation Fusion Proteins 

While ESS-specific translocations aid in the correct diagnosis and can serve as a prognostic indicator, they also constitute ideal targets for (immuno)therapy. Fusion proteins arising from chromosomal translocations represent potent tumor antigens because: (1) they are tumor-specific, (2) they are not expressed in normal tissues so there will not be peripheral tolerance towards them, (3) many of these breakpoints are shared by several patients, and (4) they are often required for tumorigenesis so they cannot downregulated in the tumors without loss of the malignant phenotype. Due to their tumor-specific nature, they can be regarded as a special class of neoantigens, which have recently been recognized as ideal tumor antigens. 

Several conditions are required for a T cell epitope to be immunogenic: (1) the protein must be present in sufficient quantities within the tumor cells, (2) the protein must be processed so that the peptide remains intact for presentation by the major histocompatibility complex (MHC), (3) the peptide needs to bind to the MHC with sufficient strength and for a sufficient amount of time, and (4) the peptide should be recognized as foreign. 

To our knowledge, nothing is known about the immunogenicity of fusion proteins arising from gene translocations in ESS tumors. However, there is sufficient proof-of-concept about the immunogenicity of fusion proteins in other sarcomas or hematological malignancies. Worley et al. described a peptide derived from the SYT-SSX fusion protein in synovial sarcoma, that binds HLA-B7 and induces specific CTLs that recognize HLA-B7-positive, SYT-SSX-positive tumor cells [30]. In the same paper, another peptide derived from SYT-SSX in synovial sarcoma and a peptide derived from EWS-ATF1 in clear cell sarcoma were found to bind HLA-B27, while a peptide from EWS-WT1 in desmoplastic small round cell tumor was found to bind HLA-A3 [30]. The BCR-ABL fusion protein in chronic myelogenous leukemia (CML) has been shown to be immunogenic, with the induction of specific T cells capable of lysing tumor cells expressing the BCR-ABL protein. The BCR-ABL fusion region antigen is presented by several HLA alleles (HLA-DRB5*0101, DRB1*1501, B*3501, and B*3503) [31,32]. The PAX-FKHR fusion protein expressed in alveolar rhabdomyosarcoma has been shown to be presented in HLA-B7, resulting in the induction of peptide-specific CD8^+^ T cells capable of killing tumor cells in an HLA-B7 restricted manner. Furthermore, the authors showed that modification of the natural epitope from PAX-FKHR resulted in an increase affinity for HLA-B7 without loss of recognition by CTL for the wild-type epitope [33]. However, another study focused on the discovery of epitopes binding to HLA-A1, -A2, -A3, -DR1, -DR4, and -DR7 within the PAX-FKHR fusion and did not find immunogenic peptides [34]. It was shown that native peptides derived from the EWS-FLI-1 type 1 fusion have a poor capacity to induce CTLs, resulting in weak CTL killing of the Ewing sarcoma family of tumors (ESFT). However, the modification of anchor residues results in strong CTL activation, strong CTL killing across a range of ESFT types, and adoptive transfer of these CTLs increased survival of mice bearing Ewing sarcoma xenografts [35]. For recognition and killing of tumor cells by the immune system, endogenous processing and presentation of translocation fusion protein-derived peptides is needed. This is exemplified by the observation that an HLA-A*0201 binding epitope from the TEL-AML1 fusion region in childhood B-cell precursor acute lymphoblastic leukemia could evoke an endogenous CD8^+^ T cell response, but it was not processed by cells or purified proteasomes. This precludes the use of this epitope as a potential immunotherapy target [36].

## 5. Immunotherapeutic Studies Targeting Translocation Fusion Proteins

To our knowledge, no immunotherapeutic studies have been performed in ESS tumors that specifically target translocation breakpoint antigens. However, we provide a summary on the available knowledge on targeting translocation fusion breakpoints by immunotherapy in other tumor types. 

The group of Kawaguchi et al. reported two clinical trials on the vaccination of synovial sarcoma patients with peptides from the SYT-SSX fusion protein. In the first study, vaccination of HLA-A24-positive patients with a 9-mer peptide spanning the SYT-SSX fusion was analyzed. Vaccinations were safe but did not induce clinical responses, and rapid disease progression was observed, so that the planned six vaccinations were only possible in 3/6 patients. Delayed-type hypersensitivity (DTH) reactions were not observed and T cell frequencies using tetramer analysis were low and appeared to increase in three patients, and decrease in 1 patient. Peptide-specific CTLs could be induced in vitro from four patients, and showed peptide-specific cytotoxic activity [37]. In the second study, vaccination of synovial sarcoma patients was performed with peptides spanning the SYT-SSX fusion, either using native peptides or peptides modified at an HLA-A24 anchor residue, both alone or mixed with incomplete Freund’s adjuvant, and followed by IFNα. Clinical findings of the latter group were slightly better compared to findings in patients receiving peptide alone. Furthermore, although measured immune responses were very low, increases in CTL frequency seemed to be higher in the groups receiving the modified peptide, which might indicate enhanced immunogenicity of the modified peptide [38]. 

Dagher et al. performed a pilot trial of vaccination with EWS-FLI-1 and PAX3-FKHR fusion peptides loaded on monocytes/immature dendritic cell populations in ESFT and alveolar rhabdomyosarcoma, respectively. However, no clinical responses were observed which is possibly due to the enrolled patient population which presented with bulky, rapidly progressing tumors and a significantly impaired immune status. These patients showed rapid disease progression, due to which most patients only received one vaccination. Immune monitoring was not consistently performed in this study [39]. In a follow-up study, the same group evaluated the effect of consolidation immunotherapy during the period of clinical remission after cytoreductive therapy in ESFT and alveolar rhabdomyosarcoma. Patients underwent apheresis prechemotherapy, and after completion of cytoreductive therapy, patients received autologous T cells, influenza vaccinations, and dendritic cells pulsed with peptides from translocation breakpoints and the E7 peptide. Cohort 1 received moderate-dose IL-2, cohort 2 received low-dose IL-2 and cohort 3 received Indinavir instead of IL-2. Toxicity was minimal and all patients showed immunity to influenza within six months after cytoreductive therapy, indicating that these patients retained the capacity to respond to vaccination. However, immune responses to the translocation breakpoint peptides occurred only in 39% of patients, and only 25% of patients developed E7-specific responses. Moreover, the immune responses were transient. Overall survival of the patients was comparable to the literature data for these tumor types, but survival did not correlate with the immune responses to peptide vaccines. Thus, improvements are needed to induce strong and sustained immune responses to tumor antigens in these patients [40].

Vaccination of CML patients with a BCR-ABL breakpoint peptide vaccine in QS-21 adjuvant was safe, well tolerated and was capable of inducing specific immune responses to the fusion peptides [41]. A review of multiple BCR-ABL vaccination studies in CML patients concluded that these studies support the immunogenicity and safety of the BCR-ABL peptide vaccination, but the benefit of this approach will remain uncertain without a randomized arm to take into account any effect of imatinib alone [42]. A recent report investigated a combined tyrosinase kinase inhibitor and T cell therapy approach, based on evidence that functional leukemia-specific cellular immune responses develop in patients receiving imatinib, and possibly act in synergy with imatinib to reach disease control. They report the feasibility of producing clinical-grade BCR-ABL specific CTLs from leukemia patients and healthy donors. Following the treatment of Philadelphia chromosome-positive acute lymphoblastic leukemia patients with autologous or allogeneic BCR-ABL specific CTLS, no major toxicity was observed, and all patients achieved a molecular or hematologic complete remission after T cell therapy upon the emergence of BCR-ABL specific T cells in the bone marrow [43]. 

## 6. Roadmap to Target Translocation Fusion Proteins in ESS by Immunotherapy 

**Figure 1:**
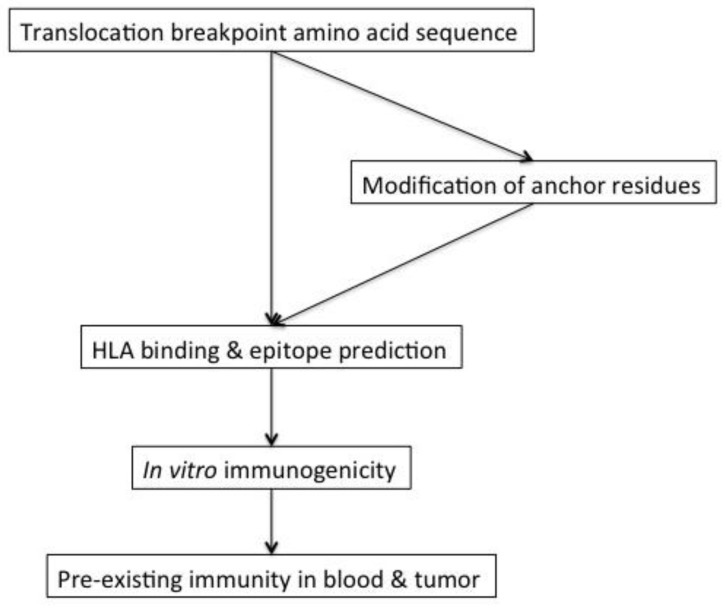
Schematic representation of immunogenicity testing of the translocation breakpoints in ESS.

The first step towards the development of immunotherapies targeting translocation fusion proteins in ESS is to evaluate the immunogenicity of the translocation breakpoints, which is schematically explained in Figure 1. This encompasses evaluation of peptide binding to HLA molecules by available epitope prediction algorithms followed by testing of the predicted peptides in in vitro immunogenicity assays. In addition, it should be evaluated whether binding of the translocation breakpoint peptides to HLA molecules can be enhanced by modifying the peptides at known anchor residues that are important for binding to the different HLA molecules without losing recognition of the endogenous epitope expressed by the tumor cells. 

Next, it should be evaluated whether there is pre-existing immunity towards these breakpoint peptides in the blood and/or tumor of ESS patients. 

**Figure 2:**
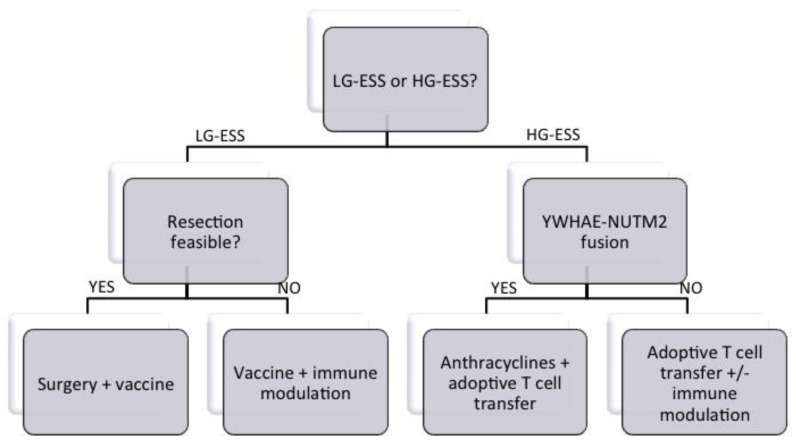
Proposed decision tree for treatment allocation to ESS patients.

Finally, the type of immunotherapy will largely depend on the patient population under study, as detailed in Figure 2. Vaccination approaches will show greater promise in patients with minimal disease (e.g., after tumor resection) or for slow-growing tumors (LG-ESS). For patients with bulky disease or with more aggressive tumors (HG-ESS), adoptive transfer of tumor-infiltrating lymphocytes or T cells engineered with a translocation breakpoint-specific TCR will be of greater value. For vaccination approaches, strategies irrespective of HLA type (i.e., covering the whole fusion region that might contain several HLA-restricted peptides) are preferred, in order to develop an off-the-shelf vaccine that is applicable in all patients carrying that translocation. Care should also be taken in selecting the vaccine adjuvant, because it is of utmost importance to induce a strong and broad immune response, preferentially both CD4^+^ and CD8^+^ T cells.

For HG-ESS tumors with the YWHAE-NUTM2 fusion, a combination of anthracycline treatment with adoptive T cell transfer might be a good choice, given the preliminary data of the sensitivity to anthracyclines of this subtype. Combination with other immunomodulatory agents might be warranted for HG-ESS, or in cases of bulk disease, to tackle tumor-induced immunosuppression. However, data on the infiltration of ESS tumors by immune cells or on the interaction between ESS and the immune system is mostly lacking. A recent study comparing ESS with undifferentiated uterine sarcomas showed that ESS contained significantly fewer myeloid cells and M2 macrophages [44]. Our own group documented high PD-L1 and B7-H4 expression in uterine sarcomas in general, but the numbers of ESS tumors (seven cases) in this study were too low to perform a subgroup analysis [45]. Thus, a comprehensive study on a sufficient amount of ESS patients is urgently needed to map the immune landscape of recurrent ESS tumors for the rational design of combination treatments. 

## 7. Conclusions

Recurrent translocations are a frequent phenomenon in both LG-ESS and HG-ESS that contribute to their tumorigenicity. These tumor-specific fusion proteins might therefore constitute ideal targets for immunotherapeutic approaches, since they are not expressed by normal cells. Although nothing is known about the immunogenicity of these breakpoint peptides in ESS, data from other tumor types are available that indicate their feasibility. Altogether, immunotherapeutic approaches targeting tumor-specific translocation fusion proteins might be promising options for recurrent ESS, provided that additional pre-clinical data about their immunogenicities will be obtained.

## Figures and Tables

**Table 1:** Immunohistochemical profiles of endometrial stromal sarcoma (ESS) tumors.

Molecule	Low-Grade ESS (LG-ESS)	High-Grade ESS (HG-ESS)	
		YWHAE-NUTM2	ZC3H7B-BCOR
CD10	++	-	+++
ER PR	+++ +++	- -	+ +
Cyclin-D1	-	+++	+++
cKIT	-	+++	unknown
BCOR	-	+++	++

**Table 2:** Genetic profiles of ESS tumors.

ESS Type	Translocation	Fusion Gene	References
LG-ESS	t (7; 17) (p15; q21)	*JAZF1-SUZ12*	[16,17,18]
	t (6; 7) (p21; p15)	*JAZF1-PHF1*	[17,19]
	t (6; 10) (p21; p11)	*PHF1-EPC1*	[19]
	t (1; 6) (p34; p21)	*PHF1-MEAF6*	[20]
	t (X; 17) (p11.2; q21.33)	*MBTD1-CXorf67*	[21]
	t (7; X) (p15; q26.1)	*JAZF1-BCORL1*	[22]
HG-ESS	t (10; 17) (q22; p13)	*YWHAE-NUTM2*	[23,24]
	der (22) (X; 22) (p11; q13)	*ZC3H7B-BCOR*	[14,25]
	t (22; X) (q13; p11)	*BCOR-ZC3H7B*	[15]
	none	*BCOR* ITD	[15,26]
